# Highly Sensitive T-Shaped Quartz Tuning Fork Based CH_4_-Light-Induced Thermoelastic Spectroscopy Sensor with Hydrogen and Helium Enhanced Technique

**DOI:** 10.3390/s24237743

**Published:** 2024-12-04

**Authors:** Yuanzhi Wang, Ying He, Shunda Qiao, Xiaoming Duan, Yufei Ma

**Affiliations:** National Key Laboratory of Laser Spatial Information, Harbin Institute of Technology, Harbin 150001, China; rwswyz@163.com (Y.W.); shundaqiao@126.com (S.Q.); xmduan@hit.edu.cn (X.D.)

**Keywords:** methane (CH_4_) detection, light-induced thermoelastic spectroscopy (LITES), hydrogen (H_2_) and helium (He) enhanced technique, T-shaped quartz tuning fork, fiber-coupled multi-pass cell (FC-MPC)

## Abstract

In this paper, a highly sensitive methane (CH_4_) sensor based on light-induced thermoelastic spectroscopy (LITES) and a T-shaped quartz tuning fork (QTF) with hydrogen (H_2_) and helium (He) enhancement techniques are reported for the first time. The low resonant frequency self-designed T-shaped QTF was exploited for improving the energy accumulation time. H_2_ and He were utilized as surrounding gases for the T-shaped QTF to minimize energy loss, thereby enhancing the sensitivity of the LITES sensor. Additionally, a fiber-coupled multi-pass cell (FC-MPC) with a 40 m optical length was utilized to improve the optical absorption of CH_4_. The frequency response of the T-shaped QTF with different concentrations of H_2_ and He was investigated, and the Q factor in the H_2_ and He environment increased significantly. Compared to operating QTF in a nitrogen (N_2_) environment, the signal amplitude was enhanced by 2.9 times and 1.9 times in pure H_2_ and He environments, respectively. This enhancement corresponded to a minimum detection limit (MDL) of 80.3 ppb and 113.6 ppb. Under different CH_4_ concentrations, the T-shaped QTF-based H_2_-enhanced CH_4_-LITES sensor showed an excellent linear response. Furthermore, through Allan deviation analysis, the MDL of the T-shaped QTF-based H_2_-enhanced CH_4_-LITES can reach 38 ppb with an 800 s integration time.

## 1. Introduction

Methane (CH_4_), as the second largest greenhouse gas in global carbon emissions, at least over a hundred years period [1], has a much higher potential threat to global warming compared with carbon dioxide (CO_2_) [2,3]. By detecting CH_4_ emissions, measures can be taken to reduce their release into the atmosphere, thereby slowing down the pace of global warming. Moreover, in production and daily life, CH_4_ has been used as one of the main fuels. However, CH_4_ is a flammable and explosive gas [4]. When it leaks into the air and encounters fire sources or electric sparks, it can cause explosions. During the production and transportation of natural gas, CH_4_ leaks not only cause resource wastage but also increase safety risks. By regularly monitoring CH_4_ leak conditions, companies can promptly identify and repair leak points, improving energy utilization efficiency and preventing accidents. Therefore, continuous and sensitive detection of CH_4_ gas is significant [5].

Various techniques have been developed for substance detection, including spectroscopic, electrical, electrochemical, microstructural, and mechanical sensing [6,7,8,9,10,11,12,13,14,15,16,17,18]. Compared with non-spectroscopy techniques, spectroscopy measurement shows merits as a reliable method for its high sensitivity, high selectivity, and quick response [19,20,21,22,23,24,25]. Spectroscopy measurement methods rely on the principle that different materials interact with electromagnetic radiation (light) in characteristic ways [26,27,28,29,30,31,32]. In the detection of gases, laser absorption spectroscopy (LAS) is a primary spectroscopic method [33,34,35,36,37,38]. The principle of laser absorption spectroscopy involves using a laser to illuminate a gas sample. Gas molecules absorb light at specific wavelengths [39,40,41]. By measuring the changes in the laser’s intensity after it passes through the gas, this technique can identify the gas components and determine their concentrations.

**Figure 1:**
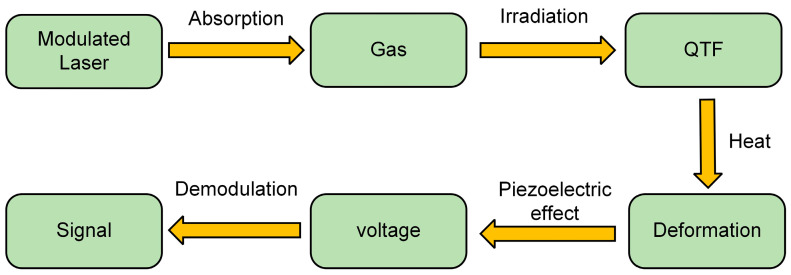
The principle of LITES.

Quartz-enhanced photoacoustic spectroscopy (QEPAS), first proposed in 2002 [42], represents a significant advancement over traditional laser absorption spectroscopy. In QEPAS, a quartz tuning fork (QTF) serves as a resonant acoustic detector. The QTF’s narrow response bandwidth allows for low background noise [43]. However, QEPAS requires the QTF to be filled with the target gas, which can be challenging in acidic and corrosive environments. In 2018, light-induced thermoelastic spectroscopy (LITES) was introduced as an innovative technique [44]. The principle of LITES is illustrated in Figure 1.

In LITES, modulated laser light passes through the gas molecules and is partially absorbed. The unabsorbed laser energy reaches the surface of the quartz tuning fork (QTF) as thermal radiation and generates a heat gradient. This heat gradient causes the QTF to undergo thermoelastic deformation [45,46]. The periodic expansion of the QTF generates a voltage through the piezoelectric effect [47]. This voltage can then be demodulated to extract signal waves, providing concentration information about the target gas [48,49]. One of the key advantages of LITES is that the QTF is separated from the target gas, enabling non-contact measurements [50]. Various methods have been reported to enhance the performance of LITES sensors, such as selecting strong absorption lines in the mid-infrared band [51], increasing the absorption path length [52,53], using custom QTFs [54], and adjusting the pressure around the QTF [55]. In 2022, an adaptive Savitzky–Golay filtering (SG filtering) technique was used in a CH_4_-LITES sensor, reaching a minimum detection limit (MDL) of 500 ppb [51]. In 2023, the first harmonic phase angle (1*f*-PA) technique combined with wavelength modulation was applied to LITES and got a 2.85 ppm MDL at 1000 s integration time [56]. In 2024, a modulation cancellation method (MOCAM) was proposed; two laser sources with optimized modulation phase irradiated on the QTF in the LITES sensor to remove the background noise and obtained a 390 ppb MDL of CH_4_ with an integration time of 50 s [57]. A Lissajous space-division multiplexed (LSDM) technology-based three-mirror multi-pass cell was presented, and a 54.8 ppb MDL in a 150 s integration time was achieved [58].

In this paper, a T-shaped QTF-based hydrogen (H_2_) and helium (He) enhanced CH_4_-LITES sensor was reported. To increase the energy accumulation time, a custom-designed T-shaped QTF with a lower resonant frequency than commercial QTFs was used as the detector. A fiber-coupled multi-pass cell (FC-MPC) with an optical path length of 40 m was employed to enhance optical absorption. Additionally, the T-shaped QTF was placed in H_2_ and He gas environments to reduce energy loss, thereby improving the performance of the LITES sensor.

## 2. Analysis of the CH_4_-LITES Sensor

### 2.1. Mechanism of H_2_ and He Enhancement

Exposing an oscillating QTF to different gas environments can cause a shift in their resonant frequency and extra energy loss, depending on gas density and viscosity [59]. The frequency shift of QTFs denoted as Δ*f* can be described by the following equation [60]:

(1) $$\frac{\Delta f}{f_{n}}=\frac{\rho_{gas}t}{2\rho h}\left(c_{1}+c_{2}\frac{1}{t}\sqrt{\frac{2\mu }{\rho_{gas}f_{n}}}\right)$$

where *f_n_* is the natural resonant frequency of QTFs; *ρ_gas_* and *ρ* are the density of gas and QTF, respectively; *h* and *t* are the width and thickness of the QTF, respectively; *μ* is the viscosity of environment gas. Meanwhile, considering the QTF with a beam resonators model [61], the Q factor, which reflects the energy loss, can be given by the following equation [62]:

(2) $$\frac{1}{Q}=\frac{3\pi \mu w+\frac{3}{4}\pi w^{2}\sqrt{4\pi \rho_{gas}\mu f_{n}}}{4\pi \rho tw^{2}f_{n}}+\frac{1}{Q_{S}}+\frac{1}{Q_{TED}}$$

where Q*_S_* is a factor characterizing the support losses and Q*_TED_* is a factor considering the thermoelastic loss. From Equation (2), it can be found that gas with smaller density and viscosity will cause less energy loss. The density and viscosity of different gases at 298 K and 1 atmosphere (atm) of pressure are listed in Table 1, according to the NIST database. Compared to the N_2_ gas environment typically used in LITES, the density of H_2_ and He reduces significantly. Meanwhile, the viscosity of these gases is relatively small. Therefore, placing QTF in H_2_ or He environments instead of N_2_ can produce less energy loss, resulting in improved signal levels.

### 2.2. CH_4_ Absorption Line Selection

**Figure 2:**
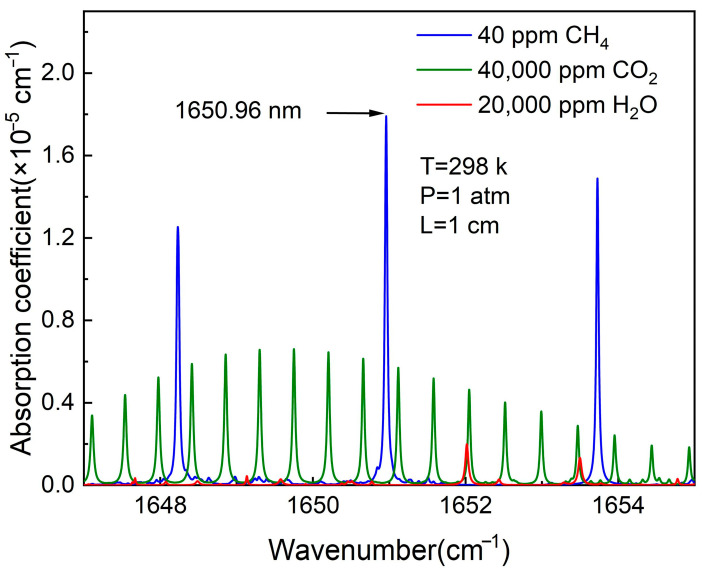
The comparison of absorption line strength of CH_4_, H_2_O, and CO_2_ at the conditions of 1 atm pressure, 297 K temperature, and 1 cm optical absorption length using the database of HITRAN.

The selection of absorption lines should adhere to specific principles. The absorption lines ought to possess sufficient absorption intensity to ensure the sensitivity and reliability of detection. At the same time, to minimize interference from background gases on the measurement results, the absorption strength of the selected lines should be significantly higher than that of any lines in the background. In addition, considering feasibility and cost-effectiveness in practical applications, the chosen lines must also be compatible with laser technologies available on the market, particularly those that can provide a stable light source and are easy to integrate. The fundamental absorption spectrum of CH_4_ is located in the mid-infrared region and, theoretically, is about two orders of magnitude stronger than overtone or combination tone absorptions. However, detection of the mid-infrared region requires expensive quantum cascade lasers. Moreover, laser technology in the mid-infrared wavelength region is not as mature as that in the near-infrared wavelength region. In this experiment, CH_4_ absorption lines in the near-infrared region were chosen for detection using a continuous wave output distributed feedback (DFB) diode laser. According to the HITRAN database, at 297 K temperature and 1 atm of pressure, the strengths of multiple gas absorption lines in the 1647 nm to 1655 nm wavelength range are calculated in Figure 2. It can be seen that CH_4_ has three absorption peaks within this range with strong absorption coefficients. Additionally, the absorption lines of water (H_2_O) and carbon dioxide (CO_2_) in this wavenumber range are distinct from those of CH_4_, thus providing good selectivity. The strongest line in the range is located at 1650.96 nm.

### 2.3. DFB Diode Laser Output Characteristics

**Figure 3:**
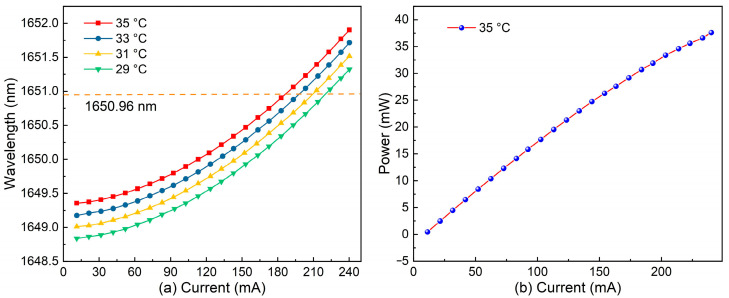
Characteristics of the DFB diode laser: (**a**) Wavelength output characteristics and (**b**) Power output characteristics.

A DFB diode laser (DFB-1650.96, Wuhan 69 Sensing Tech., Wuhan, China) with an output wavelength near 1650 nm was chosen as the optical source. The side mode suppression ratio (SMSR) of the DFB laser is 55.56 dB, indicating that the output spectrum of the DFB laser has good single longitudinal mode characteristics. The spectral linewidth of the laser is 0.11 nm. The measured performance curve is shown in Figure 3. In Figure 3a, the curves illustrate the output wavelength changes with injection current at different operating temperatures. When the laser current is fixed, the output wavelength increases with the rise in the laser’s operating temperature. When the operating temperature of the laser is constant, the output wavelength increases with the increase in the injection current. By adjusting the current and temperature, tuning of the laser wavelength can be achieved. In this experiment, the operating temperature of the laser was fixed at 35 °C, and when the injection current was set to 180.5 mA, the output wavelength matched the selected methane absorption line at 1650.96 nm. Figure 3b shows the power output characteristics of the laser at an operating temperature of 35 °C. As the current increases, the laser’s output power continuously rises. When the injection current is set to 180.5 mA, the output power of the laser reaches 29.3 mW.

### 2.4. Experimental Setup

**Figure 4:**
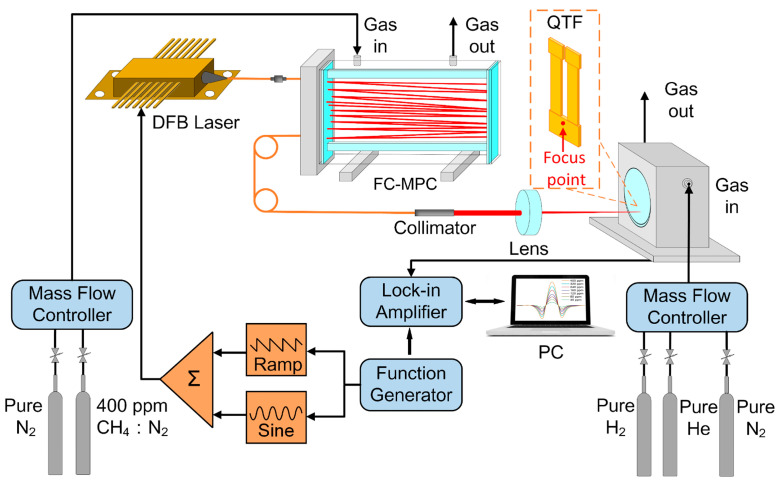
Experimental schematic of T-shaped QTF-based H_2_- and He-enhanced CH_4_-LITES sensor.

The experimental schematic of the T-shaped QTF-based H_2_- and He-enhanced CH_4_-LITES sensor is depicted in Figure 4. A DFB diode laser was used, which wavelength can be tuned. The laser beam emitted from the laser was incident into an FC-MPC through a fiber connector. The FC-MPC has a 40 m optical length to improve the optical absorption. After undergoing multiple reflections within FC-MPC, the laser beam was emitted through an optical fiber. It passed through a fiber collimator (50-1550A-APC, Thorlabs, Newton, NJ, USA) and was focused by a 50 mm lens to illuminate the surface of the T-shaped QTF. The resonant frequency of T-shaped QTF is determined as ~8675 Hz in a pure N_2_ environment, which is much lower than the 32,768 Hz of commercial QTFs commonly used. The QTF was placed in a sealed gas chamber for filling with H_2_, He, and N_2_. The system employed two gas flow controllers; one is used to adjust the concentration of the target gas, methane, while the other is used to create the gas environment of the QTF. The gases utilized include pure N_2_, pure H_2_, pure He, and a 400 ppm CH_4_:N_2_ mixture gas. To suppress background noise, wavelength modulation spectroscopy (WMS) was applied. A combination of a triangle wave and a sine wave generated by a signal generator (JDS6600, JUNTEK, Hangzhou, China) was fed into the laser controller for wavelength modulation, allowing the laser to tune its wavelength and scan the gas absorption line. A lock-in amplifier (MFLI 500 kHz, Zurich Instruments, Zürich, Switzerland) was exploited for demodulating the generated QTF piezoelectric signal. Second-harmonic (2*f*) detection was used to locate the absorption peak, and the 2*f* peak signals were utilized to determine gas concentrations. The lock-in amplifier bandwidth was set to 3 Hz. The frequency of the triangle wave is 20 mHz.

## 3. Experimental Results and Discussions

**Figure 5:**
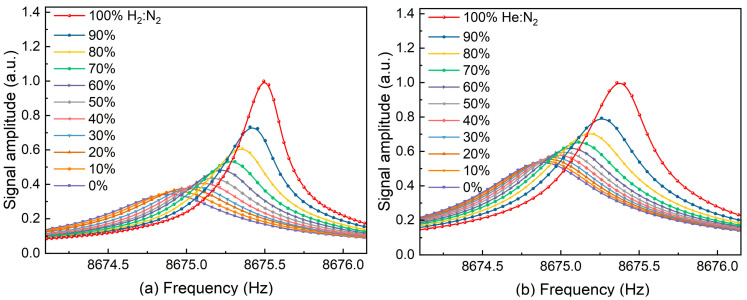
Frequency response of the T-shaped QTF with different concentrations of (**a**) H_2_ and (**b**) He.

Firstly, the T-shaped QTF was placed in different concentrations of H_2_ and He environments to study the variation in its resonant frequency and Q factor, respectively. The method of optical excitation was used to scan the T-shaped QTF frequency response curve. The concentrations of H_2_ and He were changed by controlling the relative flow rate with N_2_ using a mass flow controller. The measured frequency responses are shown in Figure 5a,b. As the concentration increased, there was a corresponding shift for the T-shaped QTF resonant frequency, accompanied by an increase in signal amplitude.

**Figure 6:**
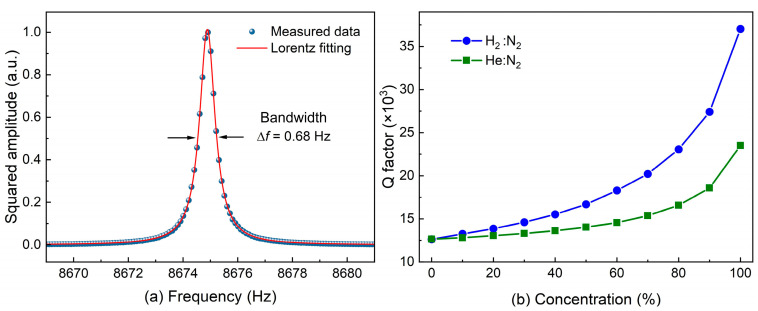
(**a**) Lorentz fitting of the T-shaped QTF (**b**) Q factor of the T-shaped QTF with different H_2_ and He concentrations.

The Q factor of a QTF can be calculated using the formula *Q* = *f*/Δ*f*, where Δ*f* represents the half-width at half-maximum (HWHM) of the QTF response, and *f* denotes the resonant frequency of the QTF. To calculate the HWHM of the QTF response, a Lorentz fitting is applied to the signal amplitudes of the QTF varied with excitation frequency. As shown in Figure 6a, the frequency response of a T-shaped QTF in nitrogen has a bandwidth of 0.68 Hz and a center frequency of 8675 Hz. Thereby, its Q factor can be calculated. Similarly, the frequency responses of the QTF under varying concentrations of H_2_ and He are fitted, and their corresponding Q factors are calculated, with the results presented in Figure 6b. The results demonstrated that the Q factor increased with the concentrations of H_2_ and He. Specifically, the Q factor for the T-shaped QTF in N_2_ was 12,654. With the addition of H_2_ and He, the Q factors improved to 23,499 and 37,031, respectively. These changes in the Q factor indicate that H_2_ and He environments lead to lower energy loss of the QTF, thereby enhancing the sensitivity of the detection system more obviously.

**Figure 7:**
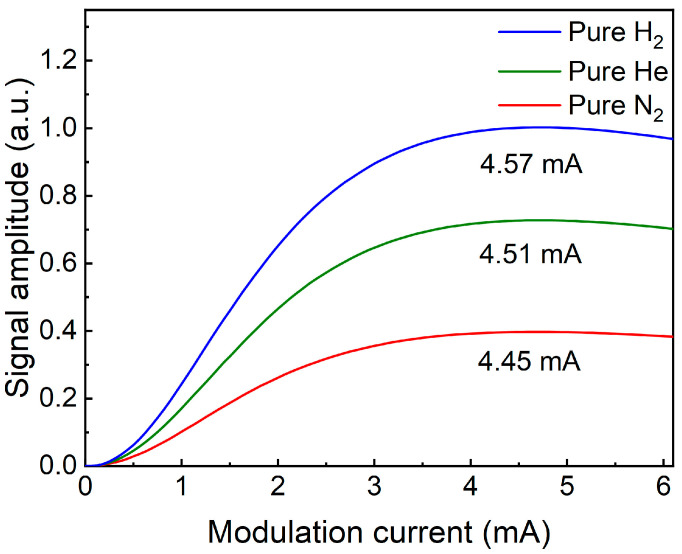
T-shaped QTF-based H_2_- and He-enhanced CH_4_-LITES sensor signal as a function of modulation current.

**Figure 8:**
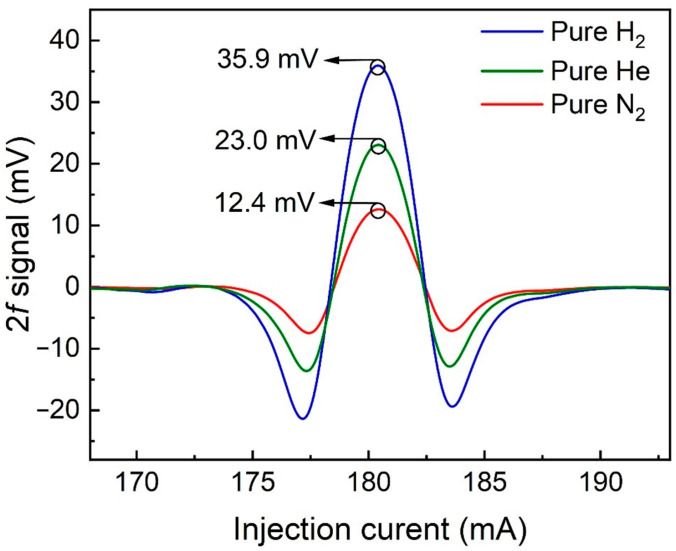
Signal amplitude of T-shaped QTF-based H_2_- and He-enhanced CH_4_-LITES sensor as a function of injection current.

The signal level was determined when 400 ppm CH_4_:N_2_ mixture gas was flushed into the FC-MPC. According to WMS, the depth of laser current modulation is correlated with the amplitude of the signal. Therefore, the modulation depth of the laser was optimized, and the signal amplitude varied from the modulation current with pure H_2_, He, and N_2_ surrounding the QTF, as depicted in Figure 7. The signal amplitude exhibited an initial increase followed by a decline in response to variations in the modulation current. The optimal modulation depth of the system is determined by the modulation coefficient of the laser and the linewidth of the absorption line. The gases surrounding the QTF influence its energy loss, so the trend of signal amplitude variation with modulation current remains consistent. However, changes in the gas environment can lead to a shift in the QTF’s resonance frequency, which in turn affects the frequency of the sine wave used for modulation. Since the modulation sine wave frequency differs, the optimal modulation current will also experience slight adjustments. The optimized modulation currents were found to be 4.57 mA, 4.51 mA, and 4.45 mA, respectively. At the same time, the laser focus position on the QTF affects the demodulated signal amplitude. By adjusting the focus position on the QTF, the maximum signal can be obtained. After the above optimization, the 2*f* signal under these three gases was measured. The results are presented in Figure 8. The peak values of the 2*f* signal were 35.9 mV and 23.0 mV when the QTF was filled with H_2_ and He, respectively. In comparison, the signal was 12.4 mV when the QTF was filled with N_2_. This shows that the signal amplitude was enhanced by 2.9 times and 1.9 times when using H_2_ and He, respectively.

**Figure 9:**
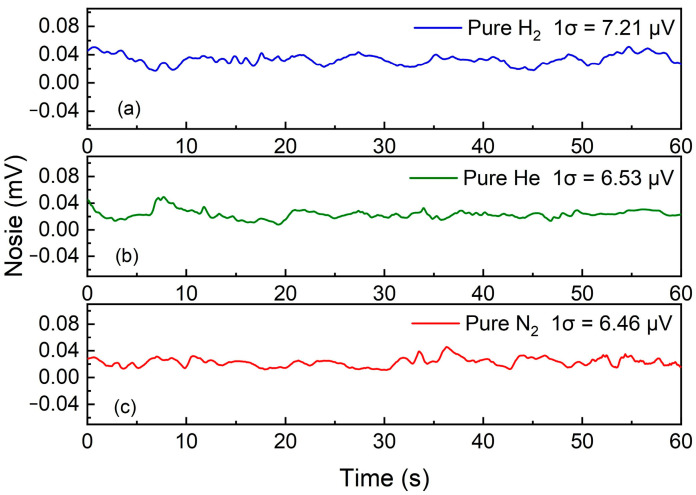
System noise measurement. (**a**) Pure H_2_ environment (**b**) Pure He environment (**c**) Pure N_2_ environment.

The noise level was quantified when the FC-MPC was charged by pure N_2_. Continuous measurement was conducted for one minute. The data presented in Figure 9 illustrate that the standard deviations (1σ) of the measured noise were 7.21 μV, 6.53 μV, and 6.46 μV for the filling gases H_2_, He, and N_2_, respectively. This indicates that filling with H_2_ and He will not have a significant impact on the noise level of the LITES system. These values correspond to MDL of 80.3 ppb, 113.6 ppb, and 208.4 ppb for CH_4_ detection. Compared to the LITES sensor filled with N_2_, the MDL of H_2_-enhanced and He-enhanced LITES sensors has improved by 2.6 times and 1.8 times, respectively.

**Figure 10:**
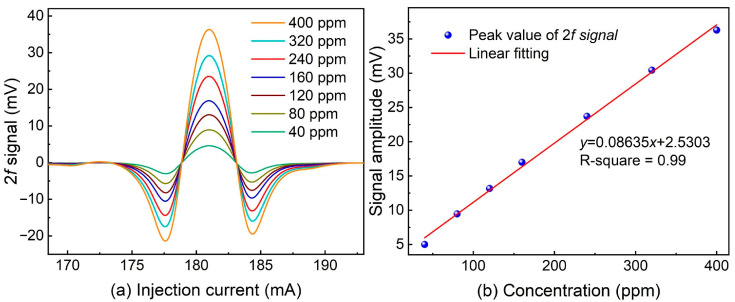
(**a**) 2*f* signal of T-shaped QTF-based H_2_-enhanced CH_4_-LITES sensor with different CH_4_ concentrations. (**b**) Linear fit of 2*f* peak signal with different CH_4_ concentrations.

H_2_ has the most effective enhancement. Therefore, the H_2_-enhanced LITES sensor system was adopted for further CH_4_ detection. To examine the linear response of the H_2_-enhanced CH_4_-LITES sensor, various concentrations of CH_4_ were prepared by diluting a 400 ppm CH_4_:N_2_ mixture with pure N_2_ using different gas flow rates. The 2*f* signal measurements obtained at these different CH_4_ concentrations are presented in Figure 10a. A linear fitting of the 2*f* peak signals is shown in Figure 10b. The results demonstrate a robust linear correlation between the 2*f* signals and the CH_4_ gas concentrations, as evidenced by an R-squared value of 0.99.

**Figure 11:**
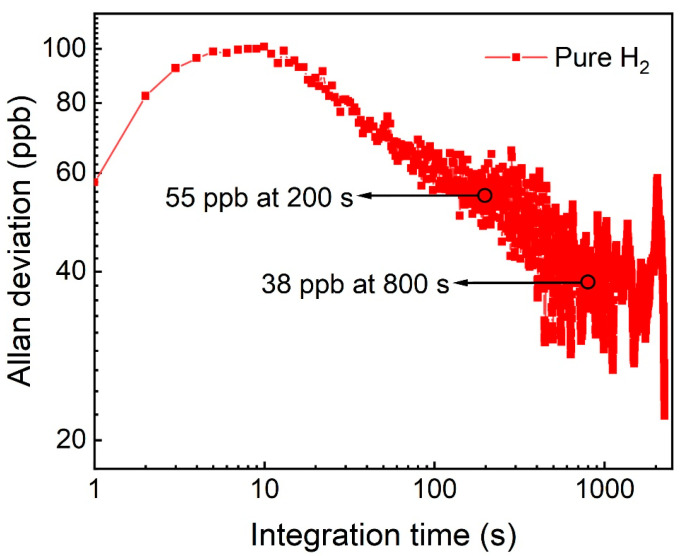
Allan deviation analysis of the T-shaped QTF-based H_2_-enhanced CH_4_-LITES sensor.

Allan deviation analysis was performed to assess the long-term stability of the T-shaped QTF-based H_2_-enhanced LITES sensor. Pure N_2_ was flushed into the FC-MPC for 2.5 h, and the results are shown in Figure 11. The analysis revealed that the MDL can be improved to 55 ppb with an integration time of 200 s, demonstrating reliable stability. Furthermore, an MDL of 38 ppb was achieved with an integration time of 800 s. Table 2 compares the MDL of the CH_4_-LITES sensor with different technologies reported to date. It is evident that the T-shaped QTF-based H_2_-enhanced CH_4_-LITES sensor presented in this work achieves the best MDL among known CH_4_-LITES sensors. This experiment provides valuable insights for the development of highly sensitive CH_4_ gas detectors.

## 4. Conclusions

In this paper, a T-shaped QTF-based H_2_-enhanced CH_4_-LITES sensor was reported for the first time. To improve the energy accumulation time, a custom-designed T-shaped QTF with a resonant frequency of approximately 8675 Hz in N_2_ was used. An FC-MPC with an optical path length of 40 m was employed to enhance optical absorption. Compared to an N_2_ environment, H_2_ and He gas environments resulted in less energy loss to the T-shaped QTF, thereby enhancing the LITES sensor’s signal level. The frequency responses and Q factors were investigated under varying concentrations of H_2_ and He. Experimental results showed that filling the environment with H_2_ and He caused frequency shifts and increased the Q factor. Specifically, placing the QTF in pure H_2_ and He environments enhanced the LITES signal by 2.9 times and 1.9 times, respectively, compared to an N_2_ environment. The MDL of the T-shaped QTF-based H_2_-enhanced CH₄-LITES sensor was 80.3 ppb, and the sensor system demonstrated excellent linear response to different CH₄ concentrations. Allan deviation analysis revealed that the MDL could be improved to 38 ppb with an integration time of 800 s, achieving a high sensitivity level.

## Figures and Tables

**Table 1:** Density and viscosity of different gases at 298 k and 1 atm.

Gas	Density (kg/m^3^) *ρ_gas_*	Viscosity (Pa·s) *μ*
H_2_	0.082658	0.87968 × 10^−5^
He	0.16414	1.9618 × 10^−5^
N_2_	1.1496	1.7573 × 10^−5^

**Table 2:** Comparison of MDL of CH_4_-LITES with different methods.

Method	Waveband	Wavenumber (cm^−1^)	MDL (ppm)	Ref.
S-G filtering	Mid-infrared	4294.55	0.5	[51]
1*f*-PA	Near-infrared	6046.95	2.85	[56]
MOCAM-	Near-infrared	6046.59	0.39	[57]
LSDM	Near-infrared	6057.08	0.055	[58]
This work	Near-infrared	6057.08	0.038	This study

## Data Availability

The data presented in this study are available on request from the corresponding authors.
